# Diseases of Animals’ Acts

**Published:** 1899-12

**Authors:** 


### DISEASES OF ANIMALS' ACTS.

The departmental committee appointed by the Board of Agriculture to inquire into the workings of the Diseases of Animals' Acts in so far as they relate to glanders, and to consider whether any more effective measures can, with advantage, be taken to prevent the spread of that disease, have presented a report containing the following recommendations:

1. That the Board of Agriculture should exercise a more extended supervision of the working of the glanders or farcy order.

2. That the glanders or farcy order should be amended to permit of notification of disease being made either to a constable or veterinary inspector.

3. That where practicable the local veterinary inspector should not engage in private practice.

4. That it should be made obligatory for veterinary surgeons to notify of cases of glanders of which they become aware.

5. That occupiers or owners of knackers' yards should notify any case of glanders found in animals taken to their yards for slaughter.

6. That horses that react to the mallein test should be considered as possible sources of infection.

7. That horses that the veterinary inspector may consider to have been exposed to contagion should be dealt with in the same manner as suspected horses, but with certain reservations.

8. That the slaughter of all animals showing "clinical" symptoms of glanders should be made compulsory.

9. That compensation for horses slaughtered solely on account of reaction to the mallein test should be on a higher scale than that for a "clinically" diseased horse, with a limit of £25. But in the event of no glanders lesions being found at the post-mortem the compensation should be the full value, with a limit of £50.

10. That the Board of Agriculture should conduct experiments with regard to the use and influence of mallein.—(*The Times*) *Veterinary Record,* August 5, 1899.

